# Particle size effect on sorting with optical lattice

**DOI:** 10.1038/s41598-020-75187-2

**Published:** 2020-10-26

**Authors:** Ebrahim Madadi, Morad Biagooi, Farhad Mohammadjafari, SeyedEhsan Nedaaee Oskoee

**Affiliations:** 1 Department of Engineering Sciences and Physics, Buein Zahra Technical University, Buein Zahra, Qazvin, Iran; 2 Department of Physics, Institute for Advanced Studies in Basic Sciences (IASBS), GavaZang Zanjan, 45137-66731 Iran; 3 Payame Noor University, Nakhl St., Artesh Blvd., Minicity, Tehran, 19395-4697 Iran; 4 Research Center for Basic Sciences & Modern Technologies (RBST), Institute for Advanced Studies in Basic Sciences (IASBS), GavaZang Zanjan, 45137-66731 Iran

**Keywords:** Applied physics, Physics

## Abstract

Transport of mesoscale particles due to driving flow fields or external forces on a periodic surface appears in many areas. Geometrical and physical characteristics of particles affect the velocities of the particles in these periodic landscapes. In this paper, we present a numerical simulation based on solving the Langevin equation for the meso-size particles subjected to the thermal fluctuations in a periodic array of optical traps. We consider the real-size particles which cause the partial trapping of particles in the optical traps. The particles are sorted for the size-dependency of particles’ trajectories. Our results are in good agreement with experiments.

## Introduction

Sorting of the mesoscale particles on a periodic two-dimensional energy landscape is of great current interest, both theoretically and experimentally. Holographic optical tweezers (HOT) are the common technique to generate such energy landscapes. Examples include the study of the transport of colloidal particles through arrays of micro-scale potential landscapes in the HOT generated potential wells^1–6^, investigation of transport and separation of overdamped particles in a microfluidic system^7^, sorting of chiral particles exploiting lattice potentials^8^, dynamic ordering and locking states of colloidal monolayers on a decagonal quasiperiodic surface^9^, sortings of particles within a microfluidic chip using a dual-channel line optical tweezers with a ’Y’ shape channel^10^ and, experimental investigation for the transport of 100 and 500 nm plasmonic nanoparticles in a two-dimensional optical lattice^11^. An array of obstacles on a surface is another technique for separating mesoscale particles^12–23^. In both cases, sorting occurs based on the fact that because of the potential wells (obstacles), particles’ path deviates from the direction of the external force (fluid flow). This deviation depends on many parameters including the strength of the potential wells (obstacles), size of the wells, external force, particle dielectric constant, shape, and size of the particles. The deviation angle in a single experimental setup depends on the characteristic properties of the particle, mostly their shapes, and sizes.

As a common method of generating HOTs, a diffractive beam splitter usually converts a single laser beam to several beams, each of which forms an individual optical trap^24^. A computer-generated hologram usually employs such a beam splitter to generate multiple traps. An array of optical tweezers can be generated in any dimension and any configuration, by making use of this method. For instance, one can refer to prismatic optical fractionation studied recently by Xiao et al.^6^. They showed that simultaneous and continuous sorting of heterogeneous samples into multiple spatially separated fractions can be done using an especial arrangement of optical traps. In addition to the collective properties of optical wells (traps), specific properties of a single well such as its width and depth can be controlled in this method. All of the above-mentioned advantages of the HOT method provide an easy fabricating device to sort an especial kind of particle.

Another method for sorting of micro-scale particles is an array of micro-scale size obstacles in the path of laminar flow of suspended particles using microfabrication techniques. As an example of such a microfabricated device, one can refer to the work by Huang et al.^12^, in which a two-dimensional matrix of silicon obstacles was employed for the separation of flexible biological molecules such as DNA without jamming or clogging. In this work, successive rows were slightly shifted laterally, which cause the fluid bifurcates flowing through the obstacles. This bifurcation of the laminar flow is asymmetric, and cause different path for particles of different size which are passing through the obstacles array. Many different papers in the literature have been devoted to the separation of particles by making use of locking states which occur on periodic surfaces. For instance, directional locking of particles in a fluid-driven through a regular lattice of cylindrical obstacles^13^, separation of drops^14^, the effects of the particle deformability on the separation of particles^17^, directional lockings in microfluidic separation systems^18^, clogging and jamming transitions for bidisperse disks flowing through a two-dimensional periodic obstacle array^25^, locking modes of the superparamagnetic beads transported in a 2D potential landscape^26^ have been studied.

Despite sorting of particles on a periodic potential landscape which is produced by HOTs, locking states have been observed in other physical phenomena. For instance, separation of particles and cells by making use of an array of slanted open cavities^16^, locking phases for the skyrmion motion on a periodic substrate^27^, phase-locking for vortex lattices on a superconducting periodic pinning array^28^, directional lockings of vortices and colloids over a square and triangular periodic substrate^29^, directional lockings of a monolayer of paramagnetic particles across a periodic substrate of a triangular lattice of magnetic bubbles^30^, have been investigated.

Many parameters affect the efficiency of sorting, including the physical properties of individual particles such as their shape and size, stiffness, and optical properties as well as their collective properties like particle density. Furthermore, the external force (laminar flow velocity), the density of traps (obstacles), and their configuration play an important role in the particle separation. The temperature plays an important role in sorting by arrays of potential wells. This is because in the case of potential wells, escaping of a trapped particle depends on the thermal fluctuations of the particle in the trap.

Since the number of parameters in the sorting process is relatively large, experimental studies can not cover all details of such a complex phenomenon, and therefore, some theoretical attempt is needed. As a preliminary effort to the theoretical study of this phenomenon, Lacasta et al. did some simulations on the classical particles sorting in a periodic potential landscape that was created by two-dimensional traps of optical tweezers^31^. They used a point-like classical particle in their simulations. The use of point-like particles causes discrepancy with the experimental results. To overcome the discrepancy with experimental results, more details are needed to take into account in the simulations. In our work, instead of considering point-like particles and modeling the size of a real particle with the width of the potential wells, we modeled the particle using a combination of point-like particles on a circumference of a circle. This can be considered as a simplified model for a spherical particle in a two-dimensional space, which our simulations were carried out. We have shown that in this case, the “*partial trapping*” phenomenon plays an important role in the sorting of particles of different sizes. The term partial trapping comes from the fact that only a portion of the particle may be affected by the potential well and as a result, the traveling particle does not trap by the well completely, however, its trajectory deflects from the original path. We have shown that partial trapping causes a notable decrease in the size of the middle plateau and make a better agreement with the experimental data.

## Methods

Lacasta et al.^31^, modeled the sorting of the particles on an array of periodic potential wells landscape. They considered a classical particle which is driven by an external force through an array of optical traps which were generated by a two-dimensional periodic function. The particle which they used in their simulations was a point-like classical particle, therefore in order to model the size of particles, they changed the width of traps. In this scenario, particles of small size were modeled by wider wells and on the contrary, narrow wells were used to model those of larger size. The governing equation of motion of the point-like particles were the Langevin equation, which includes a random force to mimic the effect of thermal fluctuations. Their results contained plateaus in the plot of absolute velocity angle for the external force angle, which belongs to the “kinetically locked-in states”. These plateaus were reported in experimental papers as well. (see, for example, Fig. 5 of Ref.^1^ and Fig. 2 of Ref.^9^) However, there is a middle large plateau in simulation results which its experimental correspondence is not nearly as large as simulation^1,9^. This incongruity was removed by increasing the temperature in Lacasta’s simulations, however, small kinetically locked-in plateaus were disappeared too, in contradiction with experiment. This discrepancy between simulation and experimental outputs can be a result of the dimensionless point-like particles which have been used in numerical simulations.

**Figure 1:**
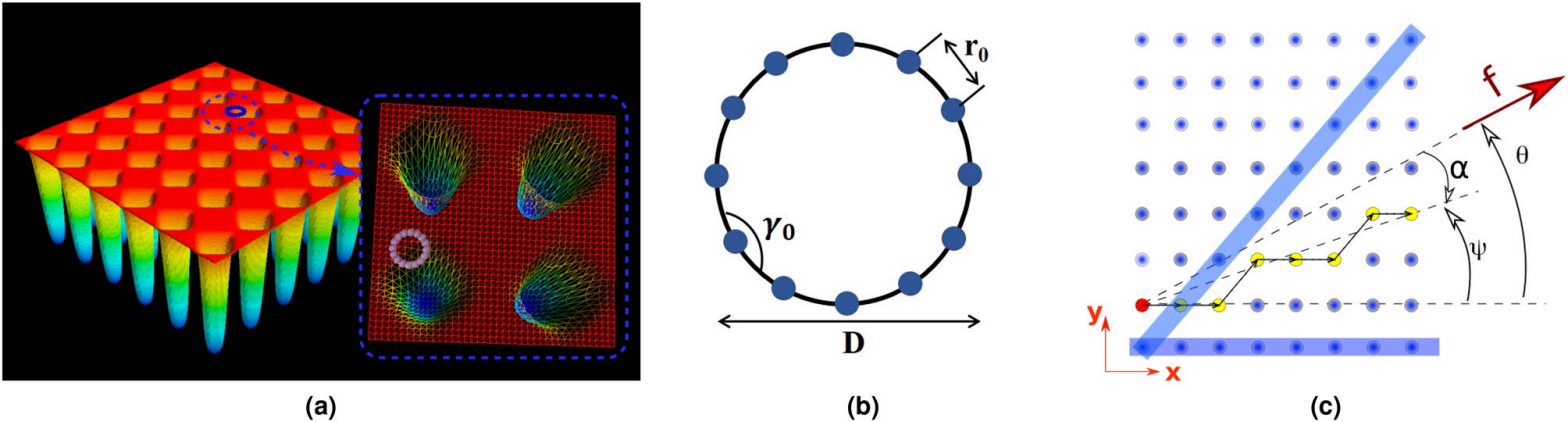
(**a**) Schematics illustration for partial trapping. As it is clear from this figure, only a small part of the particle feels potential well. This part of the figure is drawn with Mayavi^32^. (**b**) A combination of 12 point-like particles are placed together on the circumference, connected via springs to form a quasi-sphere. (**c**) Schematic representation of simulation setup. Blue strips are potential channels, as is explained in the text. ().

A point-like particle has two states when passing through the vicinity of a trap: (i) it is fully trapped by the potential well, and (ii) passing it without feeling any force. This, however, is in contrast with what usually happens in the real case, in which in addition to two above states, a particle can be partially trapped in the potential well as it is schematically illustrated in Fig. 1a. In the latter, a portion of the particle intersects with the boundary of the potential well, and as a result, depending on the size of this portion, the particle may passes the well without trapping by it. Nonetheless, the particle’s direction of motion slightly changes because the applied force from the trap, in comparison with the drag force, is not strong enough to pull the particle inside and just makes a small deviation in the particle’s trajectory.

To model the partial trapping effect, we model the suspended particle by a combination of point-like particles that are connected with elastic bands and form a quasi-sphere, as it is shown schematically in Fig. 1b. Elastic connectors obey the Hook’s law:

1 $$\begin{aligned} U(r_{i,j}) = \frac{1}{2}K (r_{i,j}-r_0)^2, \end{aligned}$$

in which $r_{i,j} = |\mathbf{r}_i-\mathbf{r}_j|$ is the relative distance between the adjacent mass of a particle, $r_0$ is the equilibrium distance between connected particles and *K* is the spring stiffness constant. To let the particle to be flexible, we considered a bending potential between adjacent pair of particles:

2 $$\begin{aligned} U(\gamma ) = \frac{1}{2} K_{\gamma } (\gamma - \gamma _0)^2, \end{aligned}$$

where $K_{\gamma }$ is the *bending stiffness constant* and $\gamma _0$ is the *equilibrium angle* between adjacent pair of particles which its numeric value depends on the initial form of the combination. In order to take the flow effect into account, each constructing particle is subjected to an external force $\mathbf{f}_i=f_0(\cos \theta _i\hat{{\mathbf{x}}}+\sin \theta _i\hat{{\mathbf{y}}})$ of a constant modulus $f_0$, which makes an angle $\theta _i$ with the direction *x* of the 2-D array of potential wells in the Cartesian coordinate system. As the original potential form used by Lacasta^31^, we modeled the two-dimensional array of potential wells by using a periodic function of characteristic length scale $\lambda$:

3 $$\begin{aligned} V(x,y) = \frac{V_0}{1+e ^{-g(x,y)}}, \end{aligned}$$

in which $V_0$ is a measure of the depth of well and $g(x,y)=A\left[ \cos (2\pi x/\lambda )+ \cos (2 \pi y /\lambda ) -2B\right]$ is a periodic function of coordinate. Parameters *A* and *B* control the steepness of the potential wells and their relative size with respect to the spatial period $\lambda$.

We used the standard Langevin molecular dynamics method to simulate the transport of individual particles. At each stage of simulation, only one particle passes through the array of traps. The governing equation of motion for each particle is as follow:

4 $$\begin{aligned} m\ddot{\mathbf{r}}_i= -{\varvec{\nabla }}_i U-\mu \dot{\mathbf{r}}_i + \mathbf{f}_i + \varvec{\xi }_i (t), \end{aligned}$$

where $U=U(r_{i,j})+U(\gamma )+V(x,y)$ is the summation of individual potentials, the subscript *i* indicates the point-like constructing particles and $\mu$ is the phenomenological coefficient of the drag force. ${\varvec{\xi }}_i(t)$ is the mutually uncorrelated thermal force acting on each particle obeying the fluctuation-dissipation relation;

5 $$\begin{aligned} \left\langle \xi _{i,\alpha }(t) \xi _{j,\beta } (t') \right\rangle = 2 \mu k_B T \delta _{ij} \delta _{\alpha \beta } \delta (t - t'), \end{aligned}$$

where the subscripts $\alpha$ and $\beta$ denote different components of the random forces, *i* and *j* indicate the individual constructing particles and $k_B$ is the Boltzmann constant.

We used the scaled variables, $\tilde{\mathbf{r}} = \mathbf{r} /\lambda$, $\tilde{t} = t \sqrt{V_0/(m\lambda ^2)}$ to make the Eq. (4) dimensionless. This reduces the number of independent variables and makes it easier to study the model. The remaining independent variables are as follow:

6 $$\begin{aligned} \begin{array}{ccc} \tilde{T} = T\frac{k_B}{\ V_0}, &{} \tilde{\mu } = \mu \frac{\lambda }{\sqrt{mV_0 }}, &{} \tilde{f}_0 = f_0 \frac{\lambda }{V_0}, \\ \tilde{K} = K \frac{\lambda ^2}{V_0^2}, &{} \tilde{K}_{\theta } = K_{\theta } \frac{\lambda ^2}{V_0}, &{} \tilde{\mathbf{r}}_0 = \mathbf{r}_0\frac{1}{\lambda }. \end{array} \end{aligned}$$

Therefore, the dimensionless equation of motion is

7 $$\begin{aligned} \ddot{\mathbf{r}}_i= -{\varvec{\nabla }}_i U-\mu \dot{\mathbf{r}}_i + \mathbf{f}_i + {\varvec{\zeta }}_i (t), \end{aligned}$$

in which we removed the tilde signs of all variables and parameters in the rest of the paper for convenience. $\varvec{\zeta }_i(t)$ is the dimensionless thermal force which obeys the following fluctuation-dissipation relation;

8 $$\begin{aligned} \left\langle \zeta _{i,\alpha }(t) \zeta _{j,\beta } (t') \right\rangle = 2 \mu T \delta _{ij} \delta _{\alpha \beta } \delta ({t} - {t}'). \end{aligned}$$

The resulting set of coupled *stochastic ordinary differential equations* (SODE) was solved using the *second-order stochastic Runge-Kutta* (SRKII) algorithm^33^. We used the Box–Muller transformation^34^ to generate the random variables with Gaussian distribution. Results are averaged over 1000 independent initial conditions and the integration was performed for $$ 10^5$$ time steps with dimensionless time step increment $$\Delta t = 0.005$$. As the original work by Lacasta, we set the numeric values of parameters $$A=5, \lambda =1, f_0=8$$ and $$\mu =20$$, which did not change during the simulation. We chose the *K* and $$K_{\gamma }$$ in such a way that the particle behaves like a rigid one. $$r_0$$ and $$\gamma _0$$ could be calculated by making use of the particle’s diameter.

In this work, we studied deviation in the direction of the velocity of the center of mass of particles from the direction of the applied force (external flow), as a function of *B* and *T*. The Cartesian components of the average velocity were evaluated in the similar method as reference^31^. $\left\langle v_{\beta }\right\rangle = \lim _{t \rightarrow \infty } \left\langle r_{\beta }(t)\right\rangle /t$, where $\beta = x,y$ and $\left\langle \cdot \right\rangle$ means average of all distinct configurations. In addition to the Cartesian components of the particles velocity, we calculate $\left\langle v_{\parallel }\right\rangle$ and $\left\langle v_{\perp }\right\rangle$, where, respectively, are the parallel and perpendicular velocity components relative to external force $\mathbf{f}$ using the following transformations:

9 $$\begin{aligned} \begin{array}{c} \left\langle v_{\parallel }\right\rangle = \left\langle v_x\right\rangle \cos \theta + \left\langle v_y\right\rangle \ sin \theta ,\\ \left\langle v_{\perp }\right\rangle = -\left\langle v_x\right\rangle \sin \theta + \left\langle v_y\right\rangle \ cos \theta . \end{array} \end{aligned}$$

Both deflection angle $\alpha$ (which is the angle of trajectory measured with respect to the applied force direction) or absolute velocity angle $\Psi$ were used to explore the deviation,

10 $$\begin{aligned} \tan \Psi \equiv \frac{\left\langle v_y\right\rangle }{\left\langle v_x\right\rangle },  \texttt {and}  \tan \alpha \equiv \frac{\left\langle v_{\perp }\right\rangle }{\left\langle v_{\parallel }\right\rangle }, \end{aligned}$$

where the absolute angle is $\Psi =\theta +\alpha$ (see Fig. 1c).

**Figure 2:**
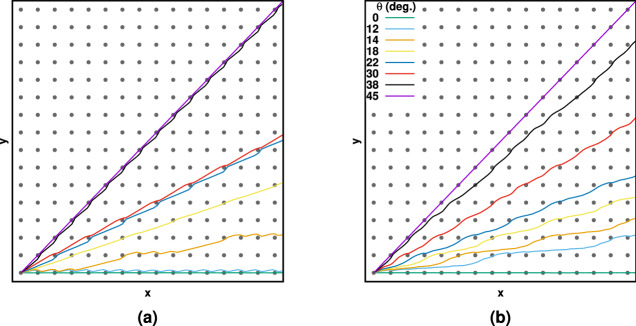
Typical trajectorys, (**a**) for a point-like particles through periodic 2D potential with parameters, $B=0.3$ and $T=10^{-4}$, and (**b**) for a real size particle with parameters $B=0.7$ and $T=10^{-4}$.

Figure 2a,b show the typical trajectories, respectively, for point-like particles and real size particles for applied force directions shown in the figure. The reason for the deviations of particle trajectories from the flow field directions is clearly shown in the figure. The particles are attracted to the traps when they are moving on the surface of the potential landscape and pull away from the force direction and the net angular displacement is observed.

## Results and discussion

**Figure 3:**
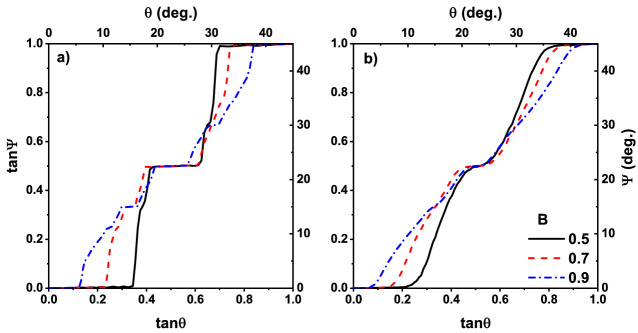
Absolute velocity angle vs. external force direction. Plateaus are observed in the absolute velocity angle for a point-like particle, for different values of control parameter B. Data in (**a**,**b**), respectively, correspond to dimensionless temperature $T= 10^{-4}$ and $T=10^{-2}$. Solid black line, dashed red line and dotted blue line, respectively, correspond to the control parameter $B=0.5$, 0.7 and 0.9. Graphs are similar to the data of Ref.^31^.

**Figure 4:**
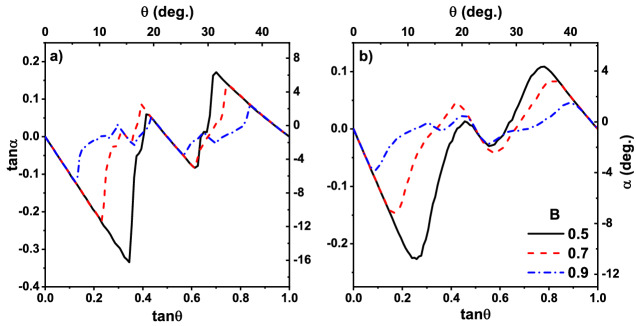
Deflection angle versus extrenal force direction for point-like particle and different control parameter *B*. (**a**,**b**) Correspond to the dimensionless temperature $T=0.01$ and $T=0.1$, respectively. Data correspond to control parameter $B=0.5$ (solid black line), $B=0.7$ (dashed red line) and $B=0.9$ (dotted blue line). Our Results are in agreement with the results of Ref.^31^.

In order to test our simulator, we first reproduced the results were reported by Lacasta setting a point-like particle where partial trapping does not happen, i. e., a traveling particle is fully trapped or escaped. Results are shown in Fig. 3. This figure is a plot of $\tan \Psi$ as a function of $\tan \theta$ for different control parameter *B*. Figure 3a belongs to the case when the dimensionless temperature is $T= 10^{-4}$ while it is $T=10^{-2}$ in Fig. 3b. All other parameters have the same numeric value as the original paper of Lacasta et al.^31^. These figures are similar to those of Lacasta, especially in the middle plateau. As it is mentioned earlier, such a large plateau has not seen in any experiment^1,9^. In addition to the absolute velocity angle, we studied the deflection angle as a function of $\theta$ which its results are shown in Fig. 4. Both cases are in good agreement with the results of the point-like particle simulation^31^.

In addition to the simulations done to explain the locked-in phenomenon, some theoretical works are done in the context of probability theory and statistical physics^35^. In this work, traps are modeled by circles with diameter *a* which their centers are located at the mesh points of a two-dimensional simple cubic lattice of lattice constant *b*. Based on the ratio of the $\eta =a/b$, authors defined two distinct regimes: A “ballistic” regime which corresponds to the dilute density of traps (small $\eta$) and a regime of packed traps (large $\eta$) which are the so called the “lattice gas” regime. Particles in this model is assumed to be point-like too, and as a result, they showed that the plot of $\tan \Psi$ vs $\tan \theta$ has several small plateaus. In order to introduce the effect of thermal fluctuations, they considered that an escaped particle has the freedom to choose its path randomly in an interval of $2\delta \theta$, centered in the flow direction. Introducing the thermal fluctuations caused the calculated results to have better agreement with experiments, however, the size of the middle plateau was much bigger than the experimental results.

Both simulation and theoretical results show that, to have more accurate results with a better agreement with experiment, we need to consider more details in modeling the sorting phenomenon. Ignoring the size of particles and considering them as point-like particles may miss some important details of the system out. To investigate the effect of the particle size on the sorting effect, we set the 16 point-like particles on the circumference of a circle with the diameter $D = 0.4$ in dimensionless scale. In this case, *partial trapping* could happen, i. e., a particle could deviate from its path by the optical tweezers if a fraction of that is illuminated by the focused laser beam.

**Figure 5:**
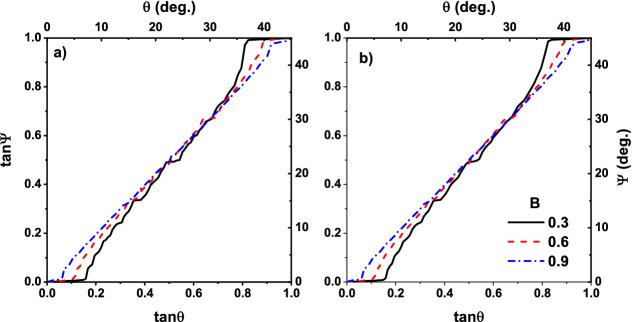
Absolute velocity angle vs. external force direction for real-size particles and different control parameter $B=0.3$ (solid black line), $B=0.6$ (dashed red line) and $B=0.9$ (dotted blue line). (**a**,**b**) Correspond to the dimensionless tempetature $T=10^{-4}$ and $T=10^{-2}$, respectively.

**Figure 6:**
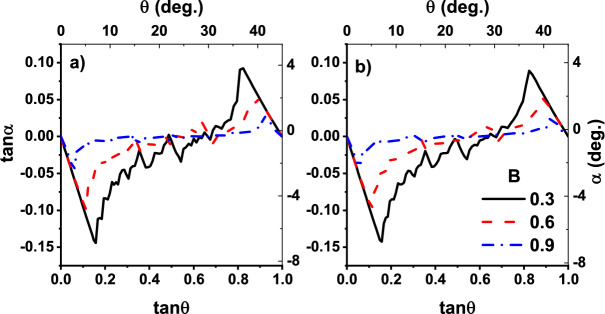
Deflection angle vs field direction for real-size particle for different control parameters $B=0.3$ (solid black line), $B=0.6$ (dashed red line) and $B=0.9$ (dotted blue line). (**a**,**b**) Correspond, respectively, to the dimensionless temperatures $T=10^{-4}$, and $T=10^{-2}$.

The results of the simulations are plotted in Figs. 5 and 6. In Fig. 5, we have plotted the absolute velocity angle $\Psi$ as a function of $\theta$ for real-size particles. Figure 5a belongs to the dimensionless temperature of $T=10^{-4}$ and the Fig. 5b is for $T=10^{-2}$. When $B = 0.3$, which belongs to the wide potential wells (traps), the resulting curve has a good agreement with those of experimental observation^1^, size of their middle plateaus decreases in both Fig. 5a,b. This is true for thinner potential wells corresponding to the control parameter $B=0.6$ and 0.9 too. In contrast, the size of first ($\tan \theta <0.2$) and last ($\tan \theta >0.8$) plateaus depends on *B*. This is due to the existence of a wider cross-section of collision in the case of a wider trap. Furthermore, as it is observed in the experiment^1^, particles are locked in the directions of [10] ($\tan \Psi =0$) and $[1\bar{1}]$ ($\tan \Psi =1$) when $\tan \theta <0.2$ and $\tan \theta >0.8$ respectively, therefore, a wider cross-section of collisions results in larger plateaus. Besides, transitions between plateaus occur in the $\tan \theta =0.3,0.5,0.6$, in agreement with experiment^1^. Directional locking observed in the experiments of separation of particles using periodic obstacles too^14,15,18,20,21^. In the paper by Devendra et al.^15^, the locking phenomenon takes place in the migration angle $\Psi \approx 0^{\circ }, 20^{\circ }, 27^{\circ }, 45^{\circ }$ for different particle sizes driven with gravity. Risbud *et al* showed that particles moving through a lattice of obstacles lock-in $\tan \Psi \approx 0, 0.2, 0.3 ,0.5 ,0.7 ,1$ directions^18^, which is consistent with the results of our simulation. In the obstacle case directional locking observes when the particles are trapped in the channels between obstacles, while for periodic potential landscape, hopping particle between adjacent traps is the main mechanism of path locking. In latter case, however, traps make a channel in direction [10] and $[1\bar{1}]$ which its width depends on *B*, as schematically represented in Fig. 1c. These trapping channels are the main reason for existence relatively large plateaus for $\theta <0.2$ and $\theta > 0.8$, particles entering the lattice in these angles, have less chance to leave the channel. This can be obtained from Fig. 6 too, where the deflection angle $\alpha$ is more pronounced for $\tan \theta <0.2$ and $\tan \theta >0.8$.

Figure 5 indicates the fact that temperature has not any significant effect on the size of the middle plateau. The curves become just smoother by increasing *T*. Indeed there is a competition between thermal fluctuations as the random force and forces imposed by optical traps. Depending on the depth of the potential well, if enough time is given to a trapped particle, thermal fluctuations provide it an opportunity to escape. By increasing temperature, thermal fluctuations strengthen or equivalently, traps become weaker. This is clear from the dimensionless form on temperature $\hat{T} \sim T/V_0$. Therefore particles have less bound to the potential wells in higher temperatures, resulting in smoother curves. Furthermore, the initial and final plateaus have a small deviation from their values at zero temperature (compare with the results of theoretical work^35^) which indicating again on the fact that our results are not sensitive to the temperature and the increasing two orders of magnitude in temperature is negligible. In addition to the middle plateau, the size of the other plateaus become more realistic in our model, especially ones that correspond to the $\theta > \tan ^{-1}(0.8)$. In contrast with point-like particle simulations, our results are more similar to the experiments. Effect of temperature also appears in Fig. 6, were increasing the temperature resulted in smoother graphs.

Increasing the numeric values of the parameter *B* causes a decrease in the width of traps. Thinner trap, weaker locked-in effect and consequently, smaller plateaus appear. On the other hand, as it is shown in both Figs. 3 and 5, by increasing *B* size of all plateaus decrease. This is another evidence that the numeric value of *B* is not a good choice for controlling the particle size, the particle’s geometry should be included explicitly in any physical simulation.

## Conclusions

In summary, we modeled a real-size particle with a combination of point-like particles on the circumference of a circle which are connected with the same springs. By solving the Langevin equation under the influence of constant external field and thermal fluctuations, we studied the transport of colloidal particles on a two-dimensional surface with periodic potential wells. This model explains those features have been observed in the experimental investigations. It was shown that the partial trapping of the particles which occurs in the array of potential wells could explain the difference between experimental and simulation results. Besides, we showed that the temperature has a negligible effect on the appearance of plateaus.

We showed that by considering the size of particles explicitly in our model, the large middle plateau which appeared in previous simulations becomes smaller and has a better agreement with experimental results. In addition we showed that plateaus corresponding to $\tan \theta > 0.8$ and $\tan \theta <0.2$ fit well with experiments.

Our work can be extended in some directions. We assumed quasi-spherical rigid particles for our simulations but this method can be used to simulate particles with more complex geometries such as elliptical shape rings. Furthermore, particles with less rigidity can be simulated with this model too, says deformable half side particles. Our model also can be extended to simulate the directional locking and sorting of Janus particles. The flexible particles called skyrmions^27^ are of current interest, thus, the model can be used to simulate the sorting of them.
